# Identification of a *TP53* Deletion in an Undifferentiated Embryonal Sarcoma of the Liver Provides Clinically Relevant Longitudinal Detection of Circulating Tumor DNA

**DOI:** 10.1200/PO.21.00102

**Published:** 2021-09-07

**Authors:** Prachi Kothari, Talia Sauerhaft, Nancy Bouvier, M. Irene Rodriguez-Sanchez, Jinru Shia, Anita Price, Sejal Morjaria, J. Theodore Gerstle, Neerav N. Shukla, Michael V. Ortiz

**Affiliations:** ^1^ Department of Pediatrics, Memorial Sloan Kettering Cancer Center, New York, NY; ^2^ Sloan Kettering Institute, Memorial Sloan Kettering Cancer Center, New York, NY; ^3^ Department of Pathology, Memorial Sloan Kettering Cancer Center, New York, NY; ^4^ Department of Radiology, Memorial Sloan Kettering Cancer Center, New York, NY; ^5^ Department of Medicine, Memorial Sloan Kettering Cancer Center, New York, NY; ^6^ Department of Surgery, Memorial Sloan Kettering Cancer Center, New York, NY

## Introduction

Undifferentiated embryonal sarcoma of the liver (UESL) is a rare hepatic tumor, with approximately 7 cases per year in the United States.^1^ Historically, these tumors fared quite poorly with a median survival of < 1 year.^2^ Advances in management of these tumors including surgical resection, with or without liver transplantation, along with adjuvant high-dose alkylator- and anthracycline-based chemotherapy provides a curative outcome in the majority of cases.^1,3-7^ Although there is no universally accepted treatment, the systemic approach is generally similar to that of other soft tissue sarcomas.^3-5,7^ Notably, a recent pooled analysis of UESL demonstrated a 5-year overall survival of 70% for those who were able to undergo surgical resection.^6^ When UESL arises in the context of a mesenchymal hamartoma, an otherwise benign liver mass, the tumor generally contains a t(11;19) (q13;q13.4) fusion.^8^ The only other established recurrent genomic finding in UESL cases lacking the fusion is the loss of function of *TP53*, evident as aberrant expression of *TP53* via immunohistochemistry.^9,10^

Plasma cell-free DNA (cfDNA) has been used for diagnostic and longitudinal disease monitoring across numerous cancer types.^11-16^ Interrogation of tumor-specific aberrations in plasma cfDNA using droplet digital polymerase chain reaction (ddPCR) has been established as a highly sensitive approach.^17-20^ As a known oncogenic driver, the ability to capture mutations in *TP53* in plasma cfDNA samples has proven to be useful for longitudinal disease evaluation and assessment of clinical outcome in multiple solid tumors.^21,22^ In this proof-of-concept case, we describe the clinical course of a woman with a metastatic UESL in which a tumor-derived somatic *TP53* deletion was detected in her cfDNA using ddPCR and longitudinal monitoring of this finding supported her complex clinical management. The patient in this case report provided consent for plasma collection and tumor profiling on an institutional-approved protocol. Consent was also obtained from the patient for publication of this case report.

## Clinical Case

**FIG 1.**
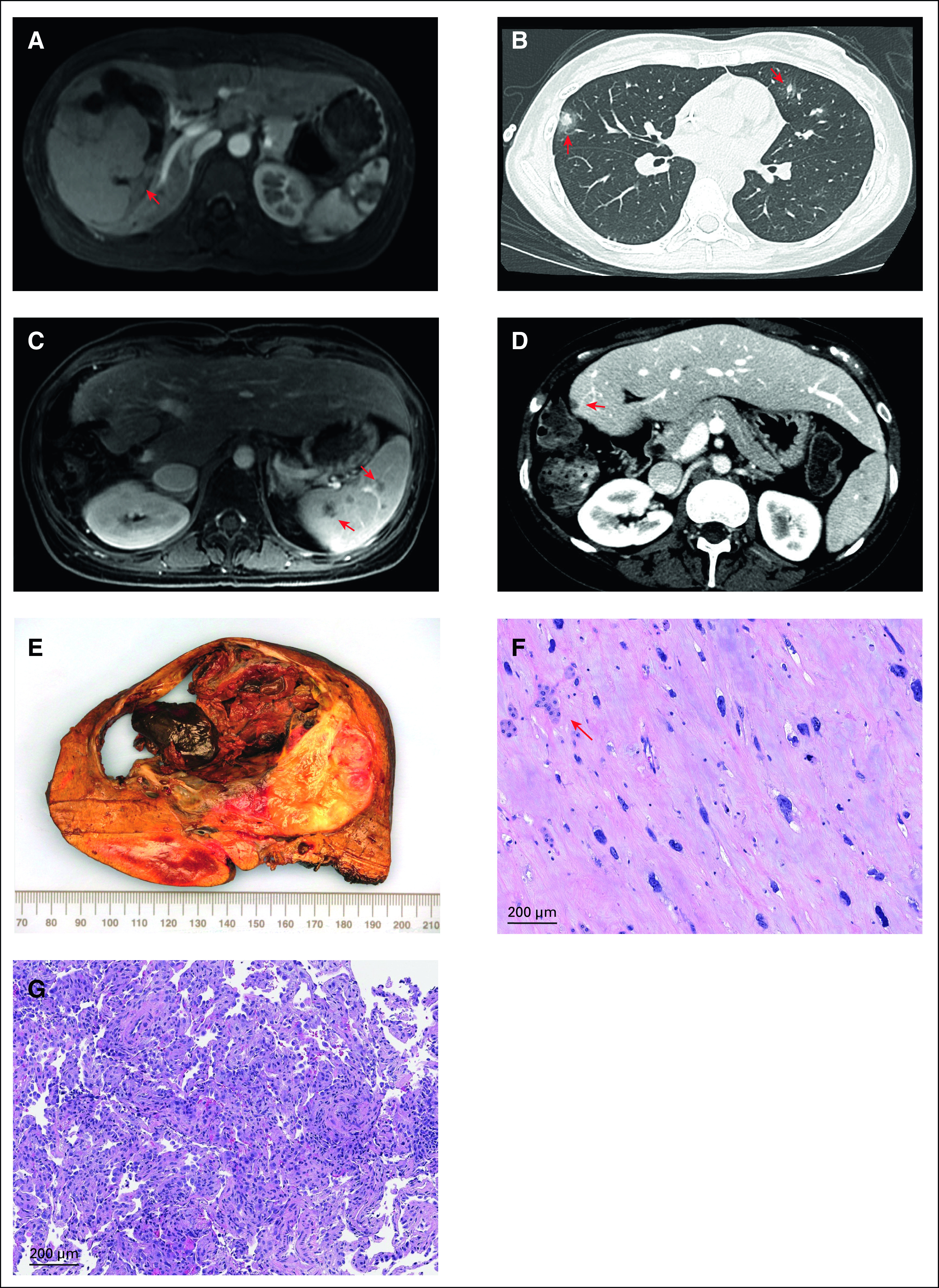
(A) MR abdomen showing liver mass at diagnosis. (B) CT chest showing lung nodules after third cycle of chemotherapy. (C) MR abdomen showing new splenic lesions. (D) CT liver showing new liver nodules. (E, F) The gross and microscopic appearance of the primary tumor. (E) Grossly, the mass is well circumscribed, compressing the surrounding liver parenchyma. The mass contains both solid and cystic components. Areas of hemorrhage and necrosis are present. (F) Microscopically, the viable tumor is characterized by spindle- or stellate-shaped cells with hyperchromatic nuclei, inconspicuous nucleoli, and ill-defined cells borders, embedded in a fibrotic matrix. Note the presence of small groups of residual benign hepatocytes (arrow). (G) The lung nodule demonstrates cellular interstitial and organizing pneumonia with no evidence of a neoplasm. CT, computed tomography; MR, magnetic resonance.

A 26-year-old woman presented with acute-onset right upper abdominal pain. Imaging revealed a 9-cm lesion in the right lobe of her liver, which was biopsied to reveal an UESL (Fig 1A). Staging demonstrated bilateral subcentimeter pulmonary nodules, concerning for metastases, which are notably significantly more prevalent at diagnosis in adults with UESL.^6^ She was treated according to our institutional approach, as previously described, initially with two cycles each containing vincristine 2 mg flat dose, doxorubicin 75 mg/m^2^, and cyclophosphamide 4.2 g/m^2^ (VDCy).^4^ Restaging revealed mild radiographic improvement, so she underwent surgical removal of the mass, which was notable for negative margins and 40% tumor necrosis (Figs 1E and 1F). Following recovery from surgery, she was treated with a third cycle of VDCy, which was complicated by a prolonged fever during her neutropenic nadir. Extensive infectious workup revealed multiple bibasilar ground glass pulmonary opacities with a halo sign, concerning for hemorrhage and/or invasive fungal infection (Fig 1B). A biopsy of the lesion revealed a cellular interstitial and organizing pneumonia, with no evidence of microbes or granulomas (Fig 1G). At this time, the differential diagnoses considered included occult infectious etiologies, particularly fungal, or possibly an idiosyncratic reaction to chemotherapy, as well as disease progression; however, this was felt to be less likely. A fourth cycle of chemotherapy was administered, including a cumulative per cycle dose of ifosfamide 14 g/m^2^ and etoposide 500 mg/m^2^ (IE). Repeat imaging demonstrated a decrease in the lung lesions but new splenic lesions. Although concerning for the possibility of occult infection or metastatic disease, a decision was made not to perform a biopsy, given the risk of the procedure and the findings from the recent lung biopsy.

**FIG 2.**
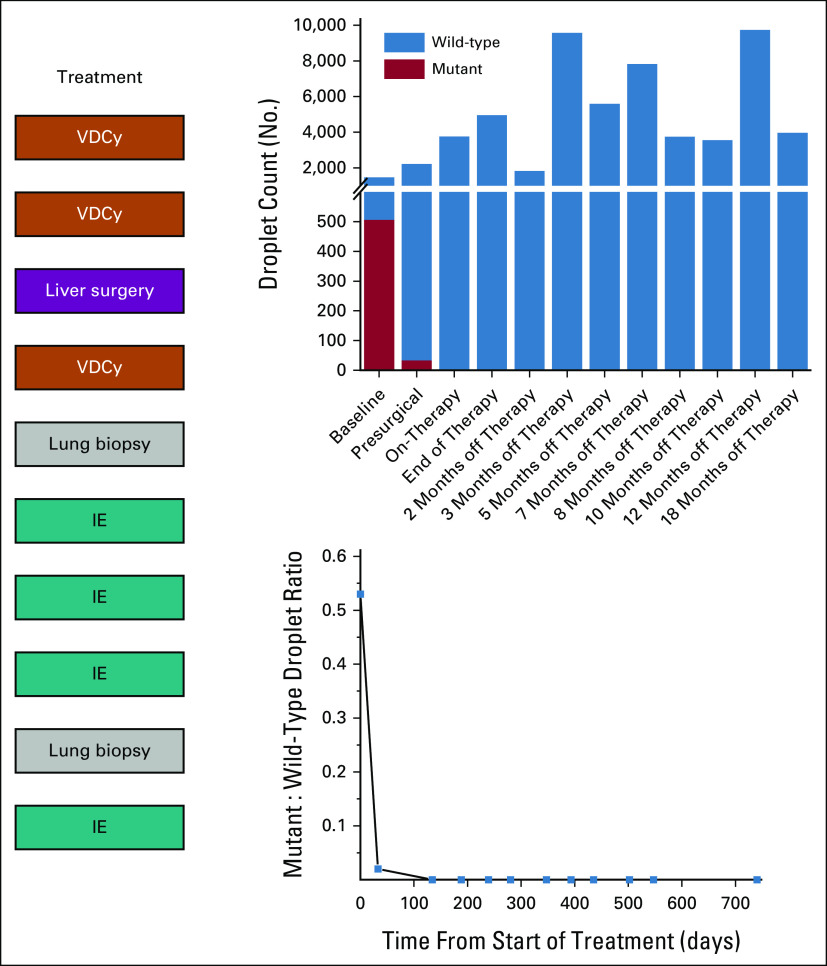
Treatment course and longitudinal *TP53* ddPCR findings from cfDNA. cfDNA, cell-free DNA; ddPCR, droplet digital polymerase chain reaction; IE, ifosfamide and etoposide; VDCy, vincristine, doxorubicin, and cyclophosphamide.

Genomic profiling of her biopsy specimen using the MSK-IMPACT next-generation sequencing platform revealed a loss of function mutation in *TP53* (deep deletion, fold change −3) and an *ATRX* M1596I missense mutation.^23,24^ We developed a specific ddPCR-based assay to detect the *TP53* deletion, which was noted between the coordinates GRCh37 17:7,538,000-7,654,000 and performed quantitative measurements of both wild-type and mutant alleles in banked plasma samples. As shown in Figure 2, although detectable at diagnosis and even before surgery, subsequent samples did not reveal any detectable mutant *TP53*. Chemotherapy was continued with another two cycles of IE, which revealed a decrease in size of the suspicious lung lesions except for a single right middle lobe lesion, which had increased in size along with enlarging splenic lesions (Fig 1C). The enlarging lung lesion was biopsied to again reveal a nonspecific inflammatory milieu, without identifiable organisms or evidence of malignancy. Because of concerns for an idiosyncratic reaction to VDCy, a final cycle of IE chemotherapy was administered.^4^ Following completion of chemotherapy, the pulmonary nodules were stable, the splenic lesions further increased in size, and there was a new isolated renal lesion. Empiric antifungal therapy with voriconazole was started. Short-interval follow-up showed unchanged pulmonary nodules and decreased size of splenic and renal lesions. The patient remained on antifungals until follow-up scans 2 months later, which showed resolution of renal lesions and stable splenic lesions at which point voriconazole was discontinued. Shortly thereafter, the patient complained of left upper quadrant abdominal discomfort, which again revealed an increased size of splenic and renal lesions. The patient resumed antifungal therapy for 10 months with generally stable findings until a new liver lesion arose just outside of the surgical field (Fig 1D). Repeat imaging 6 months later again revealed resolution of this lesion and she has since discontinued antifungals. Despite the numerous worrisome lesions, interrogation of longitudinal plasma-derived cfDNA samples for the tumor-specific *TP53* deletion has remained negative, supporting that these lesions were not indicative of sarcoma recurrence now more than 18 months off therapy (Fig 2).

## Discussion

We report the case of a young woman with UESL whose complicated treatment course was supported by molecular profiling and plasma cfDNA analysis. This is the first report, to our knowledge, of leveraging cfDNA for UESL. By identifying the tumor-specific *TP53* mutation through NGS tumor profiling of our patient's biopsy specimen, we were able to create a patient-specific ddPCR assay to follow this known driver of UESL. The absence of positive mutant droplets has provided potentially valuable clinical guidance; specifically, in terms of avoiding invasive tissue biopsies of equivocal radiographic findings.

The initial, prechemotherapy *TP53* deletion was detected at high levels, with a mutant to wild-type ratio of 0.53, which then decreased to 0.02 after two cycles of high-dose VDCy (Fig 2). The decreased *TP53*-mutant DNA circulating in her blood served as a surrogate marker of response, as corroborated by the radiographic findings and pathologic necrosis. This study highlights that when detectable, real-time monitoring of tumor cfDNA may provide an orthogonal measure to help assess response to therapy and detection of minimal residual disease. It should be noted that the fraction of mutant tumor to wild-type cfDNA may vary widely between patients and should not be, at the moment, considered a quantitative marker of residual disease, particularly as the lower limits of detection are not yet established.^25^ Furthermore, we were not able to definitively establish that this *TP53* deletion was a clonal event; however, it is likely as aberrations in *TP53* are known to be recurrent oncogenic drivers in this tumor. Therefore, an alternative explanation to our findings could have been that the mutation was subclonal, and the patient had active disease defined by non-*TP53* oncogenic drivers. Therefore, future studies should incorporate quantitative and longitudinal cfDNA measures with response to therapy and other established benchmarks, paying close attention to clonality and selecting canonical driver genomic findings for longitudinal cfDNA monitoring.

After surgical resection, this patient's cfDNA was no longer detectable; however, she developed lesions in her lungs, liver, spleen, and kidney that were suspicious for metastatic disease. Although the spleen and kidney would be unlikely areas of spread, the lungs and liver were expected sites of metastatic spread. As stated, biopsies of these samples were not consistent with malignant disease and thought to be fungal infections as they improved on antifungal therapy. The results of the cfDNA analysis were not used in clinical decision making but provided evidence that these lesions were not likely related to disease recurrence. Evaluation of plasma cfDNA during her treatment course supported the clinical decision to avoid another invasive biopsy. We were able to use the known *TP53* mutation from her tumor to track disease status from her cfDNA. She is now 18 months off therapy with no evidence of disease, both radiographically and without evidence of mutant *TP53* from plasma cfDNA.

This proof-of-concept case report demonstrates the utility of leveraging an individualized ddPCR assay to monitor tumor-derived plasma cfDNA as a noninvasive biomarker to guide treatment decisions. In this case, we were able to create a tumor-derived cfDNA test for analysis to guide clinical decisions as ddPCR has high sensitivity. However, in patients who do not have tumor tissue available for analysis, larger cfDNA panels can be used for discovery of driver mutations. For future patients, we will consider using clinically actionable quantitative cfDNA NSG panel assays for such patients.
